# Epidemiology of complete knee dislocations: an updated classification system

**DOI:** 10.1007/s00402-021-04079-5

**Published:** 2021-07-22

**Authors:** Nils Mühlenfeld, Daniel P. Berthold, Lukas N. Münch, Philipp Störmann, Jason-Alexander Hörauf, Max Leiblein, Anna Lena Sander, Johannes Frank, Ingo Marzi, Nils Wagner

**Affiliations:** 1 Department of Trauma, Hand and Reconstructive Surgery, Goethe University, Theodor-Stern-Kai 7, 60590 Frankfurt, Germany; 2 Department of Orthopaedic Sports Medicine, Technical University of Munich, Munich, Germany

**Keywords:** Dislocations of the knee, Multiligament instability, Knee surgery, Neurovascular damage

## Abstract

**Introduction:**

Current classifications of complete knee dislocations do not capture the extent of the complex concomitant ligamentous and bony injuries, which may have an impact on future outcomes. The purpose of this retrospective study was to evaluate the epidemiology of complete knee dislocations as well as to present an updated classification system based on the author’s experience at a Level-I trauma center.

**Materials and methods:**

Only patients with complete loss of contact of the articulating bones and ≥ 18 years of age who admitted in our level-I trauma center between 2002 and 2019 were included**.** Patients were identified using a retrospective systematical query in the Hospital Information System (HIS) using the International Statistical Classification of Diseases and Related Health Problems Version10 (ICD-10) codes of the German Diagnosis Related Groups (G-DRG).

**Results:**

Final data included 80 patients, with the majority of patients being male (*n* = 64; 80.0%). Mean age was 34.9 years (range: 18–70 years). External protective fixation was applied in 32 patients (40.0%). Reconstruction of the posterior cruciate ligament and the anterior cruciate ligament were performed in 56.3% (*n* = 45) and 55.0% (*n* = 44) of cases, respectively. The lateral collateral ligament complex was surgically addressed in 47.5% (*n* = 38), while the medial collateral ligament complex was reconstructed in 40% (*n* = 32). Surgery of the lateral meniscus and the medial meniscus was needed in 31.1% (*n* = 25) and 30.0% (*n* = 24). Neurovascular surgery occurred in 13.8% (*n* = 11). From the characteristic injury-patterns the authors of this study present a new classification system that ranks the injuries from Grade-A to Grade-D according to their severity.

**Conclusion:**

This retrospective study demonstrates that the historically used classification systems for dislocations of the knee are insufficient for these severe injuries. Concomitant ligamentous, neurovascular, bony, and meniscal injuries were frequent, and required several staged procedures. Consequently, an updated classification system is proposed.

## Introduction

In current orthopedic and trauma literature, knee injuries resulting in multidirectional instability are commonly referred to as “knee dislocations”, even though the articular surfaces of the joint forming bones are still in contact [1]. However, complete dislocations of the knee joint mostly occur due to intense impacts on the lower extremity as traumatic and potentially limb-threatening injuries [2]. With an incidence of under 1%, these injuries are of extremely rare occurrence [3]. Devastating consequences including loss of the extremity, chronic pain, stiffness, and recurrent joint instability along with a reduced quality-of-life may be observed, if not treated immediately and adequately. Despite the usually performed early reduction and surgical stabilization, chronic instability and stiffness of the knee remain frequent, and are associated with worse outcomes [4–6]. Besides fractures of the articulating bones, extensive damage of surrounding muscles, ligaments, menisci, and neurovascular structures may occur, requiring a sequence of complex surgical interventions.

Common historical classifications, such as the “Kennedy-Classification” or the “Schenk-Classification” consider the direction of displacement or the extent of ligament damage, instability, and presence of fracture, respectively. Interestingly, concomitant injuries of both the medial and lateral meniscus, or medial and/or lateral ligament complex are not specifically taken into account [7, 8]. Additionally, the extent of bone-damage, the presence of open dislocations, vast soft tissue damage, and neurovascular injuries are not included in these classifications. In the acute setting, however, these injuries are often referred to as one of the most important predictors for future outcomes and especially, quality-of-life [2, 9–12]. In relation to that, ligament injuries and knee instability may be rated subordinate as surgical addressing can be postponed.

Consequently, there is need for an updated classification to better reflect the exact epidemiology of knee dislocations with complete loss of contact between the articulating surfaces and concomitant neurovascular, ligamentous, or meniscal injuries. This may be of clinical relevance, as a more elaborate classification system may facilitate surgical decision-making. The purpose of this retrospective study was to evaluate the epidemiology of complete dislocations of the knee and to present an updated classification and treatment algorithm based on the author’s experience at a Level-I trauma center.

## Materials and methods

### Study design and setting

Approval from the institutional review board and ethics committee of the Goethe University medical faculty (20-881) was obtained prior to performing this retrospective study. The study followed the STROBE guidelines for observational studies (Strengthening the Reporting of Observational Studies in Epidemiology) and the RECORD guidelines (Reporting of studies Conducted using Observational Routinely collected Data) [13, 14].

A retrospective review was performed on a consecutive cohort of all patients with a complete knee dislocation at the authors’ institution between 01/2002 and 12/2019. Patients were identified via a retrospective systematical query in the Hospital Information System (HIS) using the International Statistical Classification of Diseases and Related Health Problems Version 10 (ICD-10) codes of the German Diagnosis Related Groups (G-DRG). Patients’ characteristics as well as disease-specific aspects were manually transferred from the patient’s history HIS to a digital database. All patients were checked twice in view of G-DRG code and clinical information to exclude falsely coded patients.

Patients ≥ 18 years of age were eligible for inclusion if noted to have a complete loss of contact between the articulating surfaces of the upper limb and the lower limb. In current literature, it was repeatedly demonstrated, that a large proportion of dislocated knees reduce spontaneously [15–17]. Patients were excluded from the study if it was unclear if a complete loss of contact occurred or the documentation was inconclusive. Data collection was performed with the institution’s database, and radiological examinations. Baseline demographic variables including patient age and gender were manually abstracted. Mechanism of injury was also surveyed and categorized as high-energy impact (fall from large heights (> 3 m), fall down more than five stairs, car or motorcycle accidents, bicycle accidents with velocity > 20 km/h) or low-energy impact (distortion, fall < 3 m, sports-injuries, bicycle accidents with velocity < 20 km/h, missed steps).

**Fig. 1:**
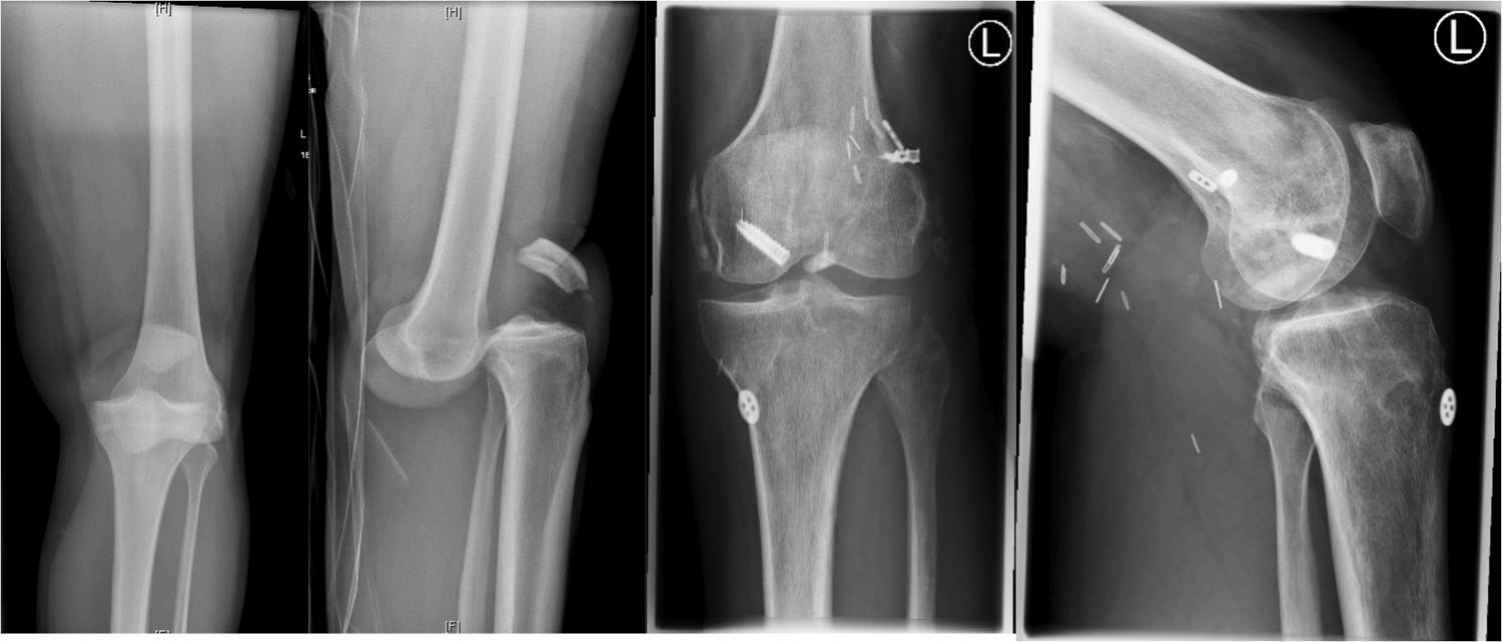
Anterior–posterior and lateral radiographs of a posterior dislocation of the knee (Grade-A) and the joint after reduction, vain graft of the popliteal artery and surgical ligament repair of the posterior cruciate ligament (PCL), the anterior cruciate ligament (ACL), and the lateral collateral ligament complex (LCL)

Patients that presented with dislocated knees upon admission in the emergency department were reduced under analgesia and conscious sedation in the emergency room or the operation theatre. In poly-trauma-patients, stabilization of the vital parameters as well as the treatment of life-threatening injuries like abdominal bleedings, thorax injuries, or intracerebral injuries were addressed first. For the better understanding of this study and better comparability, however, these injuries are not further described. After reduction of the knee, a comprehensive examination of the joint was performed under anesthesia and immobilization was applied using a cast. Evaluation of the neurovascular status was performed by clinical examination and with Doppler sonography. Patients underwent standard radiological emergency diagnosis including X-rays of the knee and computed tomography (CT) using contrast to image the blood vessels and evaluate their continuity. After emergency treatment and stabilization of the patient, Magnet Resonance Imaging (MRI) was used to assess injuries of the menisci and ligaments. Only relevant traumatic meniscus damages were included. Injuries of the medial ligament complex (MPFL, longitudinal fibers of the superficial medial collateral ligament, the deep medial collateral ligament, and the posteromedial capsule [18]) were assumed as MCL-Complex-Injuries (MCL). Injuries of the lateral ligament complex: iliotibial tract, capsular ligament as well as the posterolateral ligament complex: lateral collateral ligament; popliteus muscle and tendon; popliteal meniscal and popliteal fibular ligaments; oblique popliteal, biceps femoris tendon; arcuate ligaments, fabello-fibular ligaments; and lateral gastrocnemius muscle [19] were assumed as LCL-Complex-Injuries (LCL) (Fig. 1).

All patients were treated by orthopedic surgeons and vascular surgeons. Emergency surgery included the evaluation and reconstruction of destroyed or damaged vessels and nerves. In cases of unstable fractures, open fractures, open dislocations, or extensive soft tissue damage protective external fixation was applied. Further surgery was planned and consecutively performed in stages according to the injury pattern.

### Statistical analysis

All variables were evaluated for distribution of normality using a combination of histograms, quantile–quantile (Q–Q) plots and Shapiro–Wilk tests. Descriptive statistics were summarized as means and standard deviations for quantitative variables and as counts and frequencies for categorical variables. The significance of mean differences between continuous, normally. Differences between injuries in the impact groups were compared using chi-squared test and Fisher’s exact test. Statistical significance for all comparisons was set at *p* < 0.05. All analyses were performed with Stata statistical software (Graphpad Prism).

## Results

### Age, sex, and BMI

Of 103 patients with a coded dislocation of the knee, twenty-three were excluded from the data set as documentation was inconclusive or a complete loss of contact between the articulating surfaces was not evident. Final data included 64 men (80.0%) and 16 women (20.0%), that presented with an acute complete knee dislocation in our trauma level-I emergency department. Mean age was 34.9 years ± 13.1 (range 18–70 years) with a mean BMI of 26.8 ± 4.0 (range 22–36).

### Trauma mechanism

Most dislocated knees resulted from car accidents (*n* = 23; 28.8%), accidents with a motorcycle (*n* = 18; 22.5%) and from sports-injuries (*n* = 13; 16.3%) (Table 1). Injury resulted in 73.8% (*n* = 59) from high impact trauma, and in 26.2% (*n* = 21) from low impact trauma. The percentage of patients with concomitant meniscus damage, double meniscal damage, posterior cruciate ligament injuries, neurovascular damage, and fractures increased significantly with the intensity of the injury mechanism (*p* < 0.05) (Table 2). Differences between impact groups for the ACL, MCL, and LCL were statistically non-significant (*p* > 0.05, respectively).

**Table 1:** Distribution of trauma mechanism

Mechanism of trauma	*n*	%
Car accident	23	28.8
Motorcycle	18	22.5
Sport accident	13	16.3
Fall from height > 3 m	7	8.8
Distortion	7	8.8
Construction site accident	6	7.5
Bicycle accident	4	5.0
Fall down stairs	2	2.5

**Table 2:** The risk for meniscus injuries, posterior ligament injuries, and fractures in relationship to the impact of trauma

Impact	Meniscus (*n*)	Double meniscus (*n*)	PCL (*n*)	Fracture (*n*)
Low (21)	57.1% (12)	0.0% (0)	42.9% (9)	19.0% (4)
High (59)	83.1% (49)	37.3% (22)	71.2% (42)	47.5% (28)

### Injury pattern

**Fig. 2:**
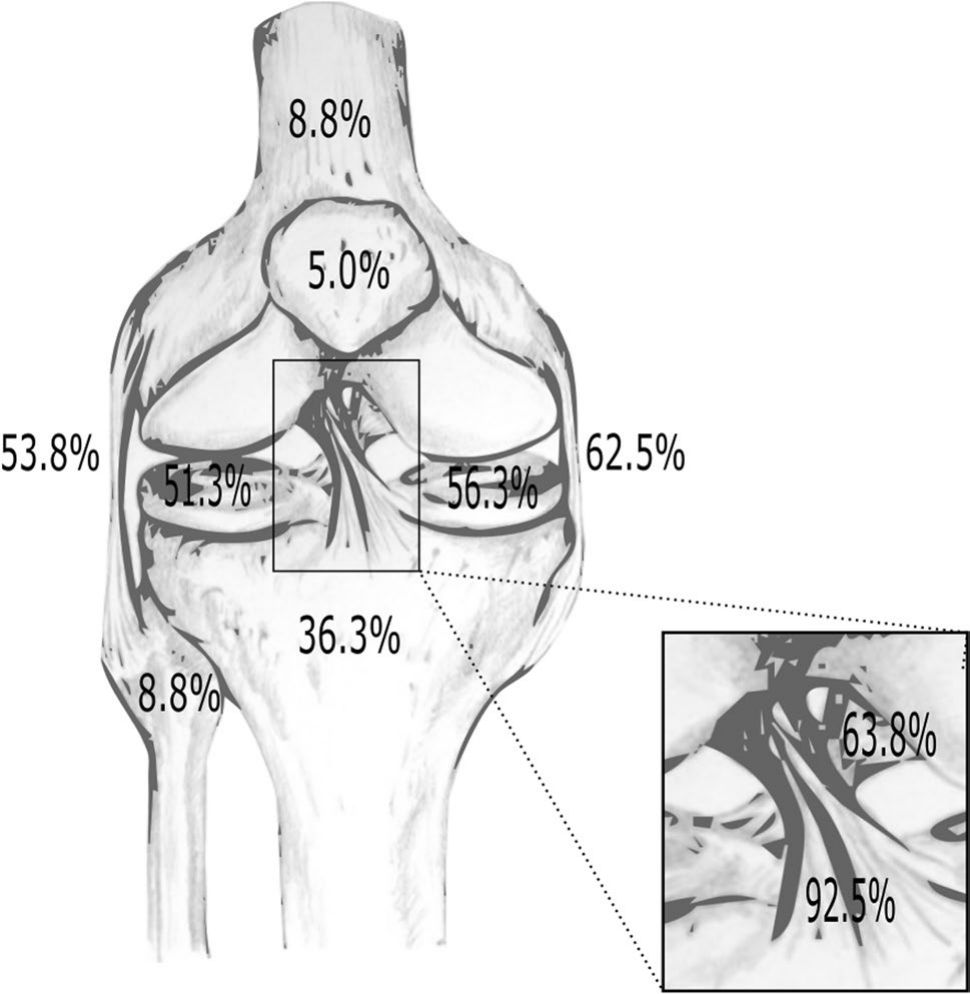
Percentage distribution of disco-ligament injuries and fractures in the present cohort of patients (*n* = 80). The box in the lower right corner shows the detailed percentage of injuries of the ACL and PCL

Most frequently documented ligament injuries were anterior cruciate ligament tears (ACLs) (*n* = 74; 92.5%), posterior cruciate ligament tears (PCLs) (*n* = 51; 63.8%), MCLs (*n* = 50; 62.5%), and LCLs (*n* = 43; 53.8%). The medial meniscus was involved in 56.3% of cases (*n* = 45), while the lateral meniscus was involved in 51.3% (*n* = 41). With 36.6% (*n* = 29), fractures most frequently occurred in the tibia (Fig. 2). A high percentage of neurovascular damages was documented (23.8%; *n* = 19). The full distribution pattern of injuries can be derived from Table 3.

**Table 3:** Distribution of injuries

Injury	*n*	%
Ligament		
Anterior cruciate ligament	74	92.5
Posterior cruciate ligament	51	63.8
Medial collateral ligament complex	50	62.5
Lateral collateral ligament complex	43	53.8
Patellar tendon	13	16.3
Quadriceps tendon	2	2.5
Meniscal damage		
Medial meniscus	45	56.3
Lateral meniscus	41	51.3
Fractures		
Tibia	29	36.3
Femur	7	8.8
Fibula	7	8.8
Patella	4	5.0
Dislocated patella	7	8.8
Neurovascular damage	19	23.8
Rupture popliteal artery	5	6.3
Dissection popliteal artery	8	7.5
Peroneus nerve damage	8	7.5

### Mode of surgical treatment

In none of the patients presented in this study, conservative treatment was indicated. The majority of patients underwent multiple surgeries (75.0%; *n* = 60), while 42.5% (*n* = 34) of patients had two, 23.8% (*n* = 19) had three, and 8.8% (*n* = 7) had at least four surgical interventions. The most repaired and reconstructed ligaments were the PCL with 56.3% (*n* = 45) and ACL with 55.0% (*n* = 44) of all cases. External protective fixation was applied in 40.0% (*n* = 32) as an emergency procedure. In case of neurovascular damage, surgery had to be performed in 57.9% of cases (*n* = 11/19) to ensure sufficient blood circulation and prevent ischemia and paresis in a primary or a secondary surgery. A vein graft for the popliteal artery was used primarily (6.3%; *n* = 5). Compartment splits were performed in ten cases (12.5%). The lateral meniscus was repaired in 23.8% (*n* = 19) and partially resected in 6.3% (*n* = 5), while complete removal was only required in one patient. The medial meniscus was refixed in 21.3% (*n* = 17), partially resected in 7.5% (*n* = 6) and had to be removed completely in one patient (Table 4).

**Table 4:** The performed procedures that were performed in this study are presented with their total numbers and their percentages in relation to the collective

Procedure	*n*	%
Ligament reconstruction/refixation		
Posterior cruciate ligament	45	56.3
Anterior cruciate ligament	44	55.0
Lateral collateral ligament complex	38	47.5
Medial collateral ligament complex	32	40.0
Quadriceps tendon	3	3.8
Patellar tendon	2	2.5
Meniscal surgery		
External meniscus	25	31.3
Inner meniscus	24	30.0
Fracture management		
Protective external fixation	32	40.0
Ilizarov fixator	3	3.8
ORIF Tibia	17	21.3
ORIF Femur	4	5.0
ORIF Patella	2	2.5
ORIF Fibula	1	1.3
Total knee arthroplasty	5	6.3
Patellectomy	1	1.3
Neurovascular surgery		
Vein bypass	1	1.3
Vein graft	5	6.3
Suture popliteal artery	1	1.3
Neurolysis	2	2.5
Sural-nerve interposal	2	2.5
Soft tissue surgery		
Wound revision	6	7.5
Compartment split	8	10.0
Split skin transplant	6	7.5
Flap plastic	4	5.0

## Discussion

The most important finding of this study is that historically used classification systems for knee dislocations do not allow for capturing the true epidemiology of these severe injuries. Complete knee dislocations with loss of contact of the respective articulating surfaces may require classification using a different system when compared to multiligamentary knee instabilities, as complete dislocations of the knee result in far more devastating injury-patterns along with potential future complications. As concomitant ligamentous and meniscal injuries were found to be frequently associated with complete knee dislocations, these lesions should be addressed accordingly, to prevent the development of chronic ligamentous instability potentially impairing functional outcomes and quality-of-life.

Only patients with an observed and documented complete traumatic dislocation of the upper thigh against the lower thigh were included in this study, while isolated multiligamentary instabilities of the knee without loss of contact between the joint articulating surfaces, as often subsumed in literature [1], were not defined as a “knee dislocation” and, therefore, excluded from the data set. In the present study, dislocations of the knee were shown to be an injury of the younger patient with a male preponderance, most of which resulted from motor-vehicle crashes, falls, and sports-injuries, which is consistent with literature [9]. The reported energy level of injury is extremely important and should be well documented and put into evaluation, as information regarding possible soft-tissue injury and the risk of vessel injury can be derived [20]. Accordingly, this study found that with a higher intensity of the injury mechanism, the risk for meniscal damage, double meniscal damage, posterior cruciate ligament injury, and fractures significantly increased. Since a large proportion of knee dislocations has been reported to reduce spontaneously [15–17], a thorough examination of the knee is crucial and should be performed by an experienced surgeon specialized in orthopedic trauma care to prevent missing severe injuries. Especially neurovascular damage, which in the present study occurred in over 20% (*n* = 19)—mostly peroneal nerve injury and injuries to the popliteal artery, can be limb threatening if undiagnosed. Besides compartment syndrome, these injuries represent the most feared direct complications and have to be treated immediately [15], as the risk for an amputation is documented in up to 86% of cases in miss-diagnosed or non-treated patients with dislocated knees [2, 9–12].

### New classification system for complete dislocations of the knee

**Fig. 3:**
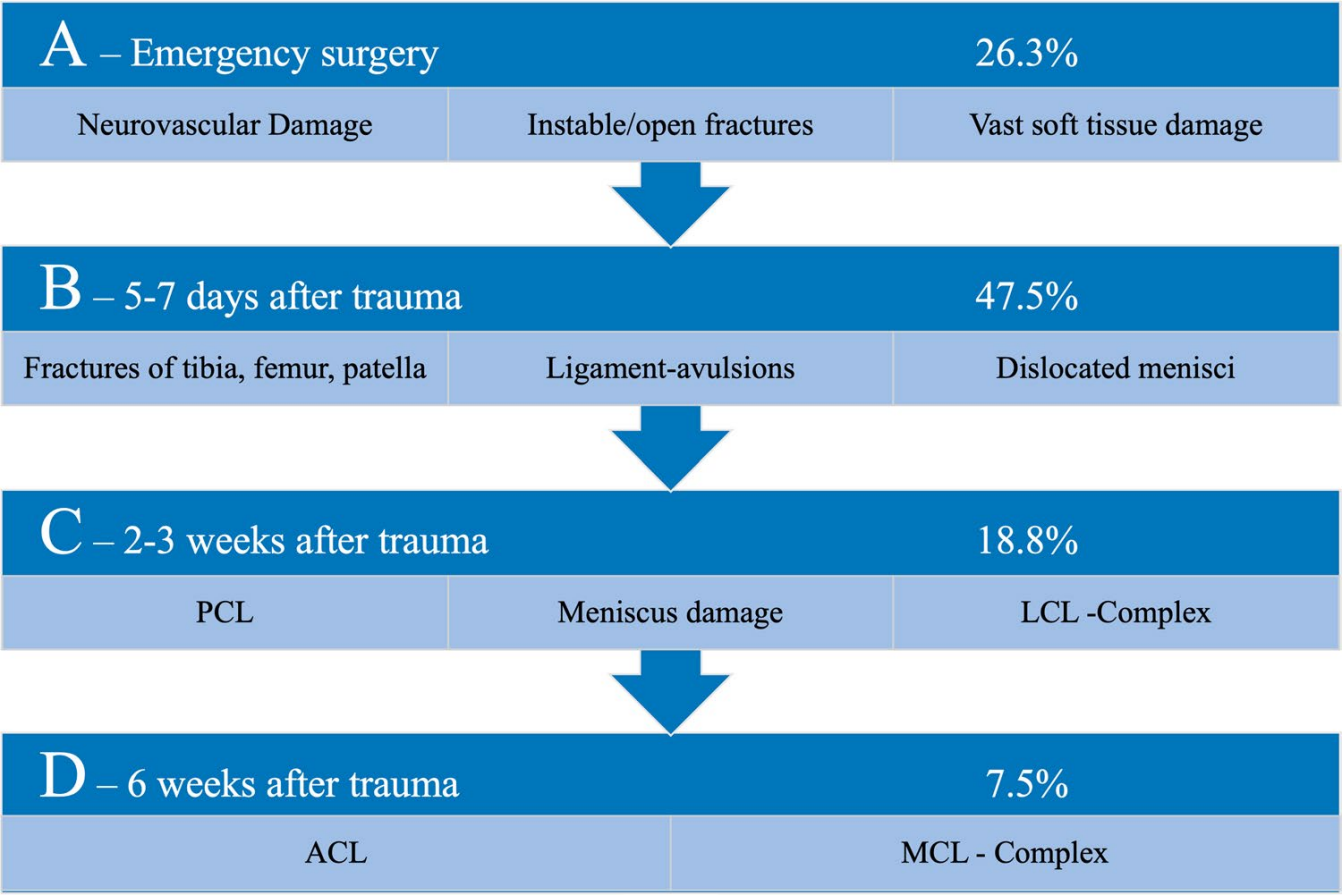
Classification-system with Grade-A to Grade-D injuries, their percentage of occurrence and the recommended time for surgery

Common classification systems like the Schenk-classification, which target only the grade of ligament instability, may not be sufficient to aid in surgical-decision making. As shown by the data of this study, the Schenk-classification does not consider the most dangerous injuries and essential outcome predicting factors. As such, it is important to evaluate the extent of ligamentous injuries [20] as well as the extent of bone and soft-tissue damage, along with the presence of open fractures or dislocations, neurovascular damage, as well as lesions of the menisci and cartilage. This approach may lead to an improved estimation for surgical urgency to preserve functionality. Consequently, based on current literature, the presented epidemiological data, and the author’s experience at a level-1 trauma center, we propose a classification system that ranks the injuries from Grade-A to Grade-D (Fig. 3).

**Fig. 4:**
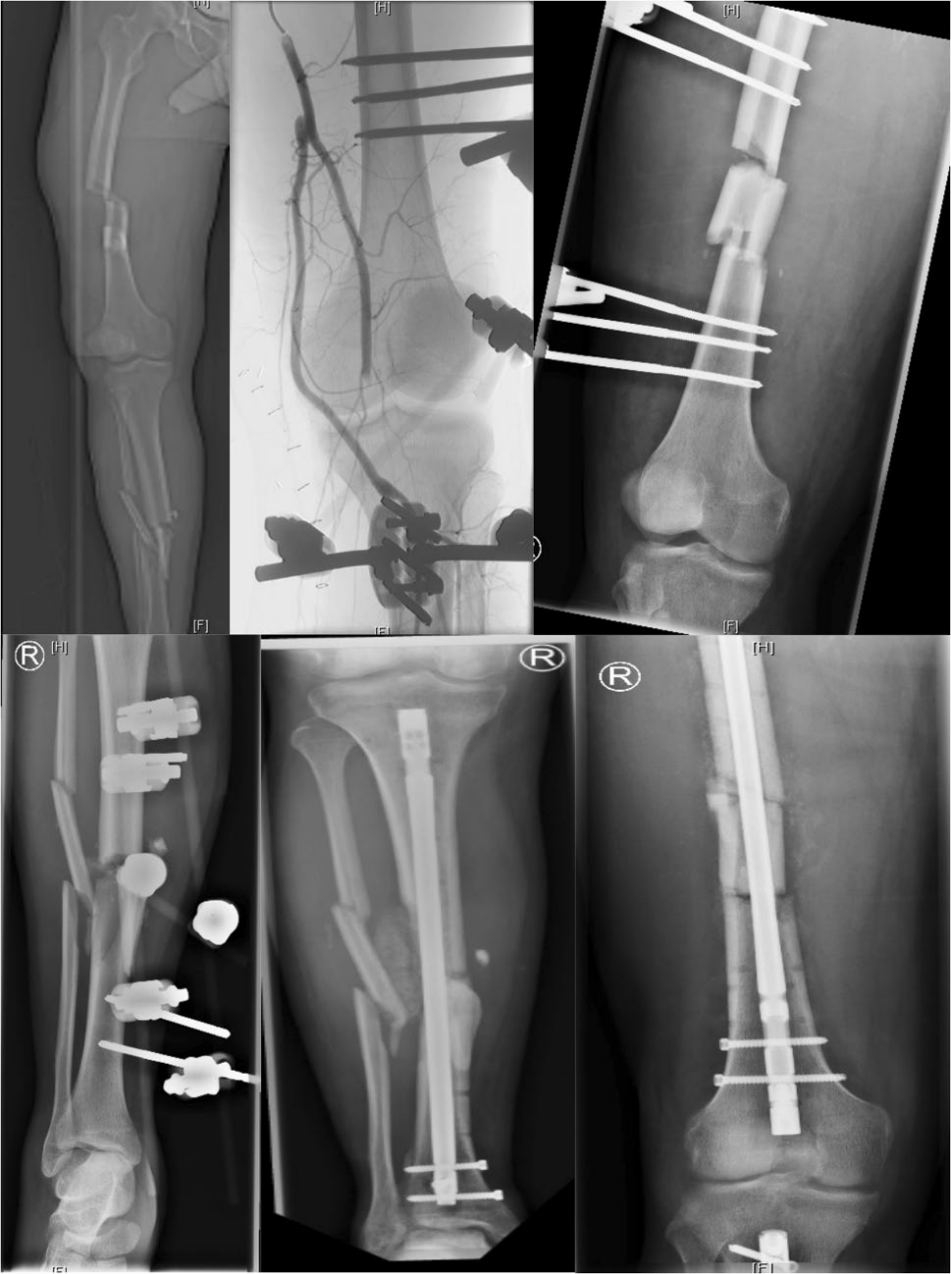
Anterior–posterior radiographs and angiography of a 32-year-old patient with a complete knee dislocation in a motorcycle accident. The figure shows the initial CT-scout of the limb, the ruptured popliteal artery as well as the leg after treatment in stages with vein graft, external protective fixation, and intramedullary nails prior to ligament reconstruction

Grade-A injuries require early or emergency surgery after stabilization of the patients vital parameters as they are factors for a poor outcome [1, 21, 22], including unstable fractures of articulating bones, open fractures, and/or open dislocations, and especially neurovascular damage. Treatment involves protective external fixation as well as soft tissue and neurovascular evaluation with potential subsequent reconstruction or transposition of nerves and vessels [22, 23] (Fig. 4). MRI-capable external fixation should be applied, or the MRI needs to be performed after its removal. In the absence of Grade-A injuries, a stable splint should be evaluated as a possible alternative.

Grade-B injuries are defined as fractures of the tibia, femur, and patella as well as ligament avulsions and dislocated menisci. Initial protective external fixation is applied. The definite reposition of fractures can be performed after 5–7 days as soon as the initial swelling has decreased to enable primary, tension-free suture of soft tissue. This includes the internal stabilization of fractures, the refixation of ligament avulsions as well as the reduction of dislocated menisci. Only few cases require complete healing in external fixation or total knee arthroplasty as rescue therapy (6.2%; *n* = 5 in this study).

Grade-C injuries include PCL- and LCL-ruptures as well as meniscus damage. The reconstruction of ligaments and menisci should be targeted as early as possible, as time of treatment plays an important role for the definite outcome, especially in axial and root tears as well as posterior horn detachments [22, 24–29]. Usually, this is the case 2–3 weeks after injury when soft-tissue injuries are healed and the associated fractures allow an arthroscopic or open reconstruction. Hereby, the PCL, which is the strongest stabilizer of the knee [30], the LCL, and meniscal injuries as well as avulsions of the ACL are generally addressed first.

Grade-D injuries comprise ACL-, and MCL-ruptures which can be treated conservatively in many cases. Reconstructions are usually performed after 6–12 weeks, when acceptable progress has been made regarding knee motion, earlier reconstructed structures had time to heal and if the patients symptoms and activity level demand further reconstruction [25, 31–33].

## Limitations

Some limitations must be considered for the present study. First, the study design was retrospective. Second, a treatment algorithm cannot be drawn from the results of this study. From our experience, in conclusion with current literature and our epidemiological data, however, we propose that stages of surgery should be selected and adapted according to the injury pattern. A definite algorithm has to be developed in the future by evaluating long-term outcomes, which were not aim of the present study.

## Conclusion

This retrospective study demonstrates that the historically used classification systems for dislocations of the knee are insufficient for these severe injuries. Concomitant ligamentous, neurovascular, bony, and meniscal injuries were frequent, and required several staged procedures. Consequently, an updated classification system is proposed.
